# Network, degeneracy and bow tie. Integrating paradigms and architectures to grasp the complexity of the immune system

**DOI:** 10.1186/1742-4682-7-32

**Published:** 2010-08-11

**Authors:** Paolo Tieri, Andrea Grignolio, Alexey Zaikin, Michele Mishto, Daniel Remondini, Gastone C Castellani, Claudio Franceschi

**Affiliations:** 1 Interdept. Center "Luigi Galvani" for Bioinformatics, Biophysics and Biocomplexity (CIG), University of Bologna, Via F. Selmi 3, 40126 Bologna, Italy; 2 Department of Experimental Pathology, University of Bologna, Via San Giacomo 12, 40126 Bologna, Italy; 3 Institute for Women's Health, University College London, Gower Street, London, WC1E 6BT, UK, and Dept. of Mathematics, University College London, Gower Street, London, WC1E 6BT, UK; 4 Institut für Biochemie, Charité - Universitätsmedizin Berlin, Charité Centrum 2 - Grundlagenmedizin, Oudenarder Strasse 16, 13347 Berlin, Germany

## Abstract

Recently, the network paradigm, an application of graph theory to biology, has proven to be a powerful approach to gaining insights into biological complexity, and has catalyzed the advancement of systems biology. In this perspective and focusing on the immune system, we propose here a more comprehensive view to go beyond the concept of network. We start from the concept of degeneracy, one of the most prominent characteristic of biological complexity, defined as the ability of structurally different elements to perform the same function, and we show that degeneracy is highly intertwined with another recently-proposed organizational principle, i.e. 'bow tie architecture'. The simultaneous consideration of concepts such as degeneracy, bow tie architecture and network results in a powerful new interpretative tool that takes into account the constructive role of noise (stochastic fluctuations) and is able to grasp the major characteristics of biological complexity, i.e. the capacity to turn an apparently chaotic and highly dynamic set of signals into functional information.

## Background - the complexity of the immune system

The vertebrate immune system (IS) is the result of a long evolutionary history and has a fundamental role in host defence against bacteria, viruses and parasites. It comprises a variety of proteins and other molecules, cell types and organs, which interact intensely and communicate in a complex and dynamic network of signals. The IS, like the nervous system, shows features of a cognitive system: it is capable of learning and memory, resulting in adaptive behaviour. Indeed, the IS creates an 'immunological memory' of previous information (primary response to a specific pathogen) and adapts itself for better recognition if the same pathogen recurs, thus providing an enhanced and more effective response. This adaptation process is referred to as *adaptive immunity *or *acquired immunity*, and makes vaccination a powerful clinical strategy [1]. Notwithstanding the availability of abundant data, a comprehensive theoretical framework for the functioning of the IS is still underdeveloped [2].

We will briefly illustrate three major conceptualizations that have been proposed to grasp the complexity of biological systems, and we will pay particular attention to the IS as one of the most complex systems in the human body, about which numerous data and several conceptualizations are already available. We will consider the concept of network [3], the functioning principle of degeneracy [4], and the recently-observed bow tie architecture [5]. Such principles are apparently quite pervasive and widespread in the organization of biological and non-biological complex systems. Several critical structures of the IS rely for their functioning on the three above-mentioned principles to afford evolvability, efficiency and robustness (i.e. non-catastrophic response to perturbation/noise) [6]. In order to point out the advantage and heuristic power of this approach, we will briefly summarize the available data on the IS as a network, and we will focus on three key immunological structures - the T Cell Receptor, Toll-like Receptor and the proteasome - to illustrate the usefulness of the concepts of degeneracy and bow tie architecture. We will finally argue that these concepts should be considered together under the perspective of a unitary hypothesis.

## The network approach

### The success of a new paradigm

Central to systems biology, the paradigm of network is also at the cutting edge of the sciences of complexity (see for example the NetSci conference series on network science at http://netsci2010.net/). Network analysis provides a powerful tool for describing complex systems, their components and their interactions in order to identify their topology, as well as structures and functions emerging from the orchestration of the whole ensemble of elements. This approach has been successfully applied to the representation and analysis of various systems in different fields, from social studies [7] to engineering and technology [8] and life sciences [3,9,10], to cite only a few examples.

The power of network conceptualization lies in the ability to grasp the characteristics of generic systems of any type, stable and physically wired (i.e. power grids, telephone/internet cabling) or dynamic and non-wired (air traffic, social networks, protein interactions). Such interdisciplinary and multi-perspective conceptualization makes it possible to consider biological systems as a whole, and to subject them to rigorous mathematical analysis.

### Networks and the immune system

Attempts to describe the IS using networks have been pioneered by Jerne [11], and have led to interesting but controversial results. This approach has recently been rejuvenated and extended by many authors with the aim of formalizing the IS more rigorously [2,12-16] within a systems biology perspective. Network models of the IS based on coupled non-linear differential equations have been used by several authors [17] and also applied to specific problems such as immunological memory [18]. This mathematical approach to the IS has also led to the proposal of IS-inspired paradigms for new types of computation algorithms [19].

Despite the above-mentioned power, usefulness and flexibility, the network approach is limited by inherent difficulties in taking into account the functional diversity of the elements and the wide (qualitative) variety of their interconnections and links, two features that strongly impinge upon the real network dynamics and behaviour of biological systems [20]. Indeed, poor characterization of the attributes of nodes and connections is a major issue in network biology. As an example, while the topological organization of metabolic networks is satisfactorily understood [21,22], the principles that govern their global functionality and their dynamics are not. Flux balance analysis of metabolism in a given *E. coli *strain revealed that network use is very unbalanced. Observations led to the conclusion that most metabolic reactions have low flux rates, but the overall metabolic activity is ruled by a number of reactions with very high flux rates. In this scenario, *E. coli *is able to react to changes in growth conditions by reorganizing the rates of given fluxes mainly within this high-flux backbone [23]. Another important issue is that network analysis is predominantly static. Multiple time points and network states can be collected and analyzed in a longitudinal fashion, but this is not yet a dynamical analysis. A further, in some ways minor, limitation may be the computational intractability of the analysis of large networks characterized by combinatorial properties. To go beyond such limits is a challenge in network theory and systems biology [3].

While the application of the network paradigm revealed the existence of *structural *complexity, many other layers of complexity in the system became apparent at the same time and evaded clearer comprehension owing to the intrinsic limitations of the network approach.

Among the principles that have been used to tackle these new levels of *functional *and *architectural *complexity, the degeneracy principle [4] and the bow tie architecture [5] have been proposed. The general consideration underlying these proposals is that biological complexity probably cannot be explained by a single concept, even a powerful one such as that of network, and that other layers of architectural complexity are present and should be identified, conceptualized and integrated.

## The principle of degeneracy

### Degeneracy is a most prominent characteristic of biological complexity

*Degeneracy *has been defined as the "ability of structurally different elements of a system to perform the same function" [4,24-26]. In other words, it refers to a partial functional overlap of elements already capable of non-rigid, flexible and versatile functionality. Consequently, a system that accounts for degenerate elements is provided with redundant functionality. Redundancy of function confers robustness, i.e. the ability to cope with (sometimes unpredictable) variations in an operating environment with minimal damage, alteration or loss of functionality. In a system composed of degenerate elements, if one fails, others can take over from it in a sort of vicarious functionality, and yield the expected output or at least a similar one (e.g. sails and oars for boat propulsion).

It is important to stress that the classical, engineering concept of redundancy is opposed to that of degeneracy, and often refers to structural similarity, repetition or multiplication. Redundancy thus refers to the *one-to-one*, or *one structure-one function *paradigm (e.g. a twin-engine boat). While redundancy in this sense can only support redundant functioning, degeneracy refers to the *many structures-one function *paradigm (the converse form of degeneracy, *pluripotentiality*, refers to the *one function-many structures *paradigm). Indeed, to make redundant use of different structures, they will be required to adapt and sustain a given function. Hence, redundant functioning of a system composed of heterogeneous elements requires degeneracy.

Within this perspective, Edelman and Gally [4] provided a list of various examples of degeneracy at different levels of biological organization: the genetic code, in which different nucleotide sequences encode the same polypeptide; the protein folding process, where different polypeptides can fold so as to be structurally and functionally equivalent; metabolism, for which multiple, parallel biosynthetic and catabolic pathways exist; immune responses, in which populations of antibodies and other antigen-recognition molecules are degenerate; connectivity in neural networks, in which there is enormous degeneracy in local circuitry, long-range connections, and neural dynamics; and many other very interesting cases.

It is to be emphasized that, as in the examples above, degeneracy is a characteristic pertaining to the elements of a system, but it impinges strongly upon the system's dynamics and functionality. Indeed, the architectural characteristics of a system and the features of individual components together play indispensable roles in forming the symbiotic state of the system as a whole and thus its dynamics [27,28].

Another structural advantage of degeneracy, in comparison to redundancy, lies in the evolvability [4,29] of the degenerate element and of the whole system. This evolutionary advantage relies on the characteristic that degenerate structures are functionally overlapping and versatile, and rearrange their configuration to meet internal or external (environmental) changes thanks to their interchangeable task capabilities. In other words, degenerate systems have a flexibility that makes them capable of yielding unforeseen functionalities, and may thus show evolutionary advantage. It is noteworthy that on a longer evolutionary time scale, this functional degeneracy coincides with the Gouldian concept of "ex-aptation": while an ad-aptation (*ad + aptus*, "shaped toward a given fitness or usage") is a feature built by selection for its current role, an ex-aptation is a character evolved for other usage (or no usage, "non-aptation") and only later - from this original usage (*ex*) - 'co-opted' for its current role [30,31].

Apart from robustness and evolvability, another intrinsic characteristic of degeneracy is the capacity to integrate different signals. There are examples of biological receptor systems that exploit this feature masterfully. In the retina of the eye, only three types of light receptors exist (one relative to each of the three fundamental colours) and they are degenerate: each is responsive to a wide range of electromagnetic frequencies (i.e. colours) and not to one precise frequency only. The integration of signals from all the degenerate receptors allows the eye to perceive an incredibly wide range of colours [26]. All these characteristics of degeneracy have long been considered fundamentally important in immunology (see Appendix for a historical perspective).

### Degeneracy in immunological structures

From a specific immunological perspective, a dynamics of the type that accounts for the retinal receptors drives the immune Toll-Like Receptors (TLRs), collectively a sort of "immunological eye", to recognize immunogenic peptides and to tune the innate immune response [13,32,33]. Each single TLR is complementary to the others, and each is able to detect a different repertoire of conserved microbial molecular patterns, so that the whole TLR system, constituted in humans by 10 different receptors [34-36], can collectively sense most if not all microbes.

It is to be noticed that degeneracy in the immunological context was originally referred to as "the ability of a single antigen to activate many different T lymphocyte clones" [4]. The T lymphocyte, or T cell, plays a central role in cell-mediated immunity, and is distinguishable by the presence of a special, hypervariable receptor on its surface (T cell receptor, TCR), which is structurally different in each cell clone. The TCR (and its co-receptors) can bind antigenic peptides presented within the groove of the Major Histocompatibility Complex (MHC) cell surface proteins, expressed by special antigen-presenting cells (APCs).

The "specificity" paradigm of the TCR has been a long-lasting concept: it was believed that each TCR could bind (and consequently initiate a response) one and one with only a specific 'cognate' antigen peptide. Mounting evidence [37] subsequently showed that a dynamics governed by the *one antigen-one antibody *rule would not have been sustainable for an organism in terms of mass, energy and response time. Today, while it is clear that the TCR maintains exquisite specificity in recognizing and distinguishing antigens, there are unquestionable proofs of TCR degeneracy as an inherent feature essential for sensing the whole antigenic peptide universe [38,39]. In this perspective, TCR degeneracy can be considered an architectural and functional property that gives rise to an optimized trade-off for reasonably full coverage of the whole potential set of antigenic epitopes [38].

## The bow tie architecture

**Figure 1:**
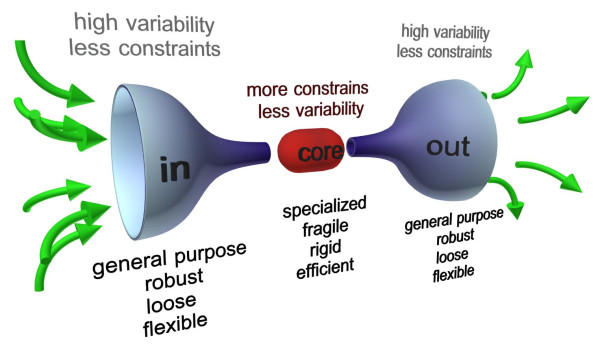
**Schematic representation of a general bow tie architecture**. Input signals conveyed through the fan in (left) are widely diversified. The capacity to admit this variability confers flexibility and robustness on the system. Then, in the core, inputs (and information complexity) are 'compressed' by relatively rigid rules and protocols, and processed into basic modular building blocks. In the core, critical decisions about the sorting and the fate of the system outputs are taken. Finally, again through protocols, a variety of elaborated output fans out, and the complexity of the original, uncompressed information is restored. Output → input feedback loops may also occur.

The "bow tie" architecture (so called for its shape; Figure 1) is a recent concept that tries to grasp the operational and functional architecture of complex and self-organized systems, including organisms. In the most general terms, bow tie architectures refer to ordered and recurrent control system structures that underlie complex technological or biological networks and are capable of conferring a balance among efficiency, robustness and evolvability. Conversely, it has been argued that the bow tie structure shows critical weak points [5], which could explain the concomitant characteristic of biological systems, i.e. their fragility towards specific evolved agents [13].

**Figure 2:**
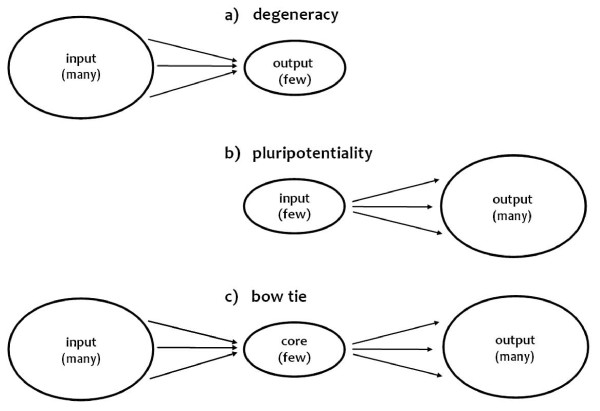
**Degeneracy, pluripotentiality and bow tie**. The concept of bow tie integrates the concepts of degeneracy and pluripotentiality: figuratively, a bow tie structure (*many-few-many*) (1c) appears from the overlapping of degeneracy (*many-to-one*) (1a) and pluripotentiality (*one-to-many*) (1b).

A bow tie architecture shows the ability to accept a wide range of inputs (in Figure 1 the left, input wing) and convert them to a reduced set of universal building blocks (the knot, or core). Here, assembly protocols act on these basic modular building blocks, eventually restoring and fanning out a wide variety of outputs (the right bow). It is interesting to note that the bow tie can be interpreted as the combination of two degenerate systems coupled through a single central element, suggesting that the two concepts of degeneracy and bow tie share a similar conceptual and architectural design, i.e. the *many-to-one *(degeneracy) and *one-to-many *(pluripotentiality) paradigm (Figure 2).

**Figure 3:**
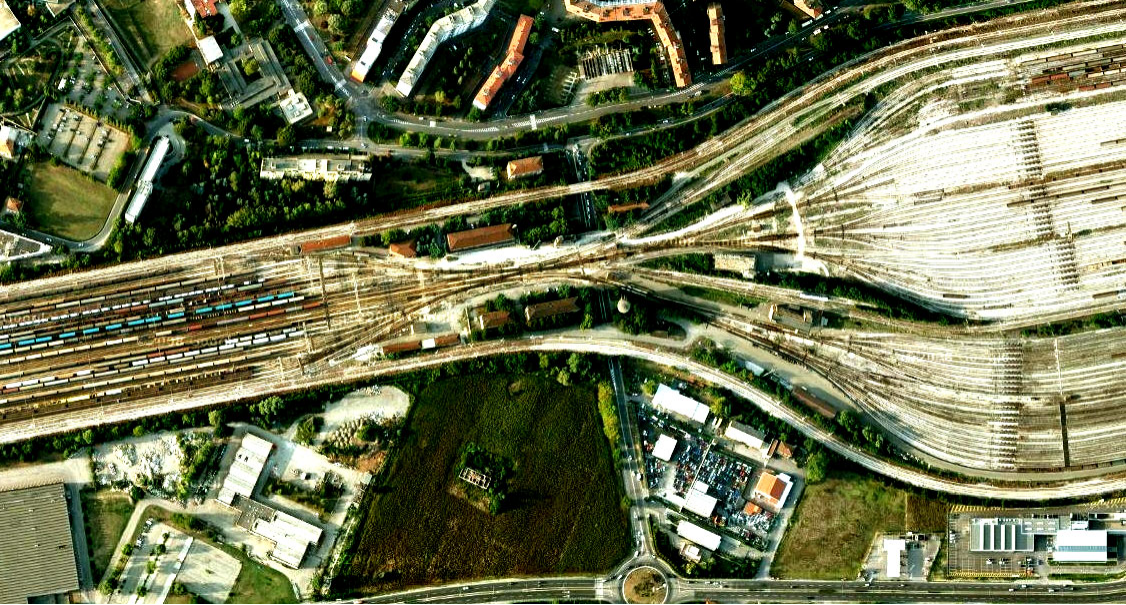
**Example of a technological structure organized as a bow tie**. Aerial view of the Bologna freight marshalling yard, clearly showing a structure analogous to a bow tie. Wagons arrive from a variety of sources (left bow); to facilitate control and sorting out operations, they are driven through a narrowing: few rails under strict supervision to ensure the maximal capability for control and decision-making; from here they are dispatched to a plethora of new destinations (right bow). Again, the narrowing (the 'core' surveillance station) allows economical and effective regulation to be taken and exercised on a variety of inputs (train provenances) and to yield a quantity of outputs (new destinations). Inspired by Needham [122], p. 170, Figure forty five. Image from Google Maps.

This kind of architecture has been observed in the structural organization of organisms throughout the biological scale as well as in technological and dynamical systems where the management, control and restriction of incoming inputs become central, e.g. metabolic networks [5,40,41], signalling networks [42], TCR signaling [6], pathways of oxygen signalling and energy of the hypoxia-inducible factor cascade [43], the Internet [44], large technological installations (see Figure 3); it also accounts for the dynamics of socio-political phenomena [45], so it may be considered wide-ranging [5].

In general terms, bow ties seem to have evolved specifically to deal with a highly fluctuating and "sloppy" environment (represented by the fan in bow) and thus to organize fluxes of information (or matter) optimally into their overall structure. Indeed, in biological systems, the metabolic process shows nested bow tie structures [5,40,41]. A large number of different nutrient inputs are catabolized ('fan in') to produce few carriers (i.e. ATP, NADH and NADPH) and just 12 precursor metabolites (pyruvate, fructose 6-phosphate, etc.), which are in turn synthesized into ~70 larger building blocks (nucleotides, amino acids, fatty acids and sugars). The building blocks then fan out into the assembly of larger macromolecules following general-purpose polymerase processing [5,40]. Thus, in metabolic networks, the core of the bow tie seems to comprise a densely connected, small-world network, which is resistant to single component failure.

The efficacy, success and observed universality of such architecture rely on its functional organization. Bow ties are able to ensure a virtually unlimited scalability, thanks to the ability to accept an incredibly high number of different inputs and, at the same time, to guarantee robustness and evolvability. Indeed, building blocks are modular (functionally independent) and can be recombined and reused through universal protocols to meet the demands of a rapidly changing environment. The core of the modular 'common currencies' facilitates system control, dampening the effects of noisy context and thus reducing fluctuations and disturbances.

Conversely, the same efficient architecture may be prone and vulnerable to fragilities due to specific changes, perturbations, and focused attacks directed against the core set of building blocks and protocols. If a hijacking process can take control over a protocol or other elements in the core, the whole system can collapse under the breakdown of its key regulatory mechanisms, or can be forced to 'execute' processes harmful for the system itself.

## Results and discussion - towards an integrative perspective

### TLR integrated functioning

Bow tie architectures have been observed in the functional structure of some key components of the innate immune response, such as the human TLRs system, and of the adaptive immune system, such as the TCR.

Even if microbial stimulatory molecules, sensed by the TLRs, constitute a very complex stereochemical set (in number and quality), and although the response involves many genes, signals mediated by the TLR system cross a funnel of diminished or compressed complexity [32], as in a bow tie core. Indeed, while the whole universe of microbial peptides can amount to more than 1000 different molecules, the TLR ligands are a reduced set amounting to > 20 elements, which can be sensed by a set of ~10 TLRs. Each TLR must thus show a degree of degeneracy [34]. Signals detected by TLRs are then mediated by very few (four) adaptor molecules, primary (two) and secondary (≈ 10) kinases, that are able to pass the signal to transcription factors (NF-κB and STAT1) which in turn can activate a large number of genes (> 500) and initiate subsequent events (> 1000) [32].

In a further analysis [13], a comprehensive TLR signalling map shows that the whole network can be roughly divided into four possible subsystems, the most important being the main system with MyD88-IRAK4-IRAK1-TRAF6 hub proteins as a bow tie core process. This core is able to mediate the activation of NF-κB and the mitogen-activated protein kinase (MAPK) cascade, which in turn activates many target genes. Interestingly, recent network topology studies highlighted that the dynamics of MAPK signalling is ruled by the pervasive presence in the cascade network of bifan motifs [46], which occur when signals from two upstream molecules *integrate *to modulate the activity of two downstream molecules. Bifan motifs are also overrepresented in transcriptional networks [47].

Unlike metabolic networks, signalling networks show a bow tie core composed by very few key molecules such as cyclic adenosine monophosphate (cAMP) and Ca^2+ ^in G-protein coupled receptor signalling [48], and MyD88 for TLRs [13]. Such signalling networks may thus be prone to fragilities owing to the perturbation of such molecules. Indeed, knockouts of such hub proteins in mice are fatal to the organism because they impair the correct signalling of the innate immune system leading to severe failures to detect pathogen-associated molecular signatures [6].

### TCR, degeneracy, bow tie and noise

Like the TLRs, the TCR system functioning resembles a bow tie, as already described by Kitano and Oda [6]. This signalling system senses and controls the critical flux of information from outside to inside the T cell using few components and protocols [6]. Thanks to its characteristic degeneracy, the TCR is able to discriminate among a larger number of ligands than any other known receptor systems (the fan in; [38]). To manage the complexity of inbound signals, the TCR molecular structure works like protocols for ligand recognition and signal transduction. These protocols operate at the level of the single receptor as well as at the emerging level that derives from integration of multiple signals by the collective of interacting cells. The signal originating from ligand binding is a function of the affinity of the TCR for peptide-MHC complexes and of their concentration [49]. The TCR machinery is thus able to decompose and translate it into TCR signal strength, which finally determines the various cell functional outcomes. This condition determines a continuum of inputs to the TCR ("TCR signalosome") and is atypical among cell receptors, requiring elaborate computational capabilities by the TCR system [49].

There are other interesting features in the TCR architecture: the TCR machinery shows a characteristic modular design in terms of functional and spatial separation of its ligand-binding modules lacking intrinsic signalling capability [50]. Moreover, owing to exposure to continuous, weak TCR-ligand interactions, the TCR works under 'noisy' conditions. In this respect, there is now mounting evidence that this noise has a functional role in terms of receptor sensitivity: non-activating TCR-ligand interactions may modulate the sensitivity of T cells to antigens [51].

All these advanced characteristics (diversification of inputs, protocols for complex signal integration/transmission, modular design, functional noise) can be framed and fully understood only through the simultaneous consideration of more than one powerful yet single concept such as that of degeneracy. This integrative approach is not only able to explain a complex set of features, it also opens unanswered questions regarding the composition of the TCR bow tie core, the impact of TCR bow tie core proteins on global TCR dynamics, and the comprehension of TCR signal processing protocols.

### Proteasome: packing principles into a single chamber

Other crucial IS structures that show bow tie architecture are proteasomes, organelles constituted by large protein complexes with the main function of degrading unnecessary or damaged proteins by proteolysis. They are highly polyspecific enzymes because they are able to process a wide range of cellular proteins. Through the available proteasome machinery, a single cell is able to collect 2 × 10^6 ^proteins per minute, which are degraded by the physical chamber formed by the complex of 14 distinct protein subunits, working under well-specified protocols for protein degradation. The degradation core then fans out ~10^8 ^oligopeptides per minute [52]. Several isoforms of proteasomes with slightly different specificities are present, often at the same time, in a single cell [53,54]. The ratios among different proteasome isoforms could be modulated by various factors and are proposed to play a role in several diseases [55-59]. One of these isoforms, known as the immunoproteasome, enhances the generation of specific antigenic epitopes that are presented to the MHC class I molecules on antigen-presenting cells and recognized by CD8+ T cells. In an informational sense, the proteasome can be considered as a signal processing system: it processes a protein, cleaving it into peptides, which may be further cleaved in single amino acids by aminopeptidases or transported into the ER and exposed as epitopes on MHC class I complexes [60]. In the latter case, proteasomes 'extract' more epitopes from the single amino acidic sequence of the original protein (the antigen), which could activate several CD8+ T cell clones (*one-to-many*). Intriguingly, two different groups have discovered in recent years that the proteasome-mediated "sequence extraction" from a given antigen could result from a splicing of two non-contiguous sequences [61]. Very recent investigations suggest that this phenomenon, called proteasome splicing, is not a rare event and therefore represents an example of further pluripotentiality because it provides more epitopes from a given antigen than canonically supposed [62]. Therefore, within proteasome-mediated MHC class I antigen presentation, two antithetic principles could be recapitulated: the pluripotentiality of proteasome-mediated epitope production (pluripotentiality further expanded by proteasomal splicing), followed by the degeneracy of CD8+ T cell activation mediated by the MHC class I - epitope signal. Indeed, epitopes extracted from a given antigen have different amino acid sequences and could lead to the activation of different CD8+ T cells; these latter then recognize the single antigen and, as a consequence, the correlated pathogen. This concurrence of pluripotentiality and degeneracy is probably the most important attribute of the cell-mediated immune response and it allows the IS, for example, to struggle against the high mutability of virus.

### Proteasome, bow tie and noise

Certainly, as signal processing system, the proteasome operates under the action of a fundamental biological condition: noise. As stochastic fluctuations in the quantitative parameters that rule the functioning of living systems at diverse levels [63], noise is present in each stage of proteasome function. There are two aspects of signal processing under noisy conditions. First, the system should be robust against noise and fluctuations and be able to respond to the noisy signal. Second, the system, owing to evolutionary adaptation, may have evolved to use noise for *constructive *purposes. We believe that the robustness of operation of the proteasome in performing sequence-specific protein cleavage is provided by the digital nature of the amino acid sequence. This excludes the influence of noise in the sequence; however, noise is still present in the fluctuating quantity of protein copies and, as thermodynamic noise in the course of protein binding to the proteasome, in protein translocation and binding to the cleavage terminal. Could this noise counter-intuitively play a constructive role and not corrupt the quality of signal processing? In statistical physics, four basic noise-induced phenomena are known, each leading to noise-induced ordering of a non-equilibrium system. These basic effects are stochastic resonance [64], noise-induced transport [65], coherence resonance [66], and noise-induced phase transitions [67]. It is important to note that noise-induced phenomena have been experimentally detected at all levels of biological functionality, e.g. in plankton detection by paddle fish [68], in the human balance system [69], in the retrieval processes of the human memory [70], and in human brain waves [71]. Even more importantly, it has been shown that biological systems may evolutionarily adapt so that the intensity of noise is optimal for the mechanisms behind noise-induced phenomena. How can noise potentially play a constructive role in proteasome function? Some authors have addressed the question whether protein translocation inside the proteasome chamber can be driven by fluctuations and have derived a toy-model to show that translocation is probably based on a fluctuation-driven transport mechanism [72]. At the moment, there is no experimental verification of this hypothesis; however, we expect that this could be obtained if the translocation function were reconstructed from the experimental data using the method suggested by Goldobin et al. [73]. On the other hand, considering the proteasome as a signal detection system, it would be logical to assume that the detection is evolutionarily optimized to use the principle of stochastic resonance. Stochastic resonance has manifested itself as a generic phenomenon widely found in biological systems. One more argument in favour of this hypothesis is that proteins dealing with responses to external changes are much more noisy in terms of their concentration, as for example those involved in intracellular protein synthesis. This follows from the proteomic analysis and reconstruction of biological noise [63]. Signal detection in the form of epitope extraction occurs in much more noisy conditions such as simple protein digestion, so it was evolutionary profitable for proteasome function to be optimized to this genetic noise.

## Conclusion and perspectives

The increasing awareness that biological complexity is not satisfactorily described by widely-used but single and isolated concepts drives the quest for integrative theoretical scaffolds to achieve a more comprehensive, systemic understanding of biological systems, including the IS. It is crucial, in this perspective, to clarify the structure-function relationships of biological systems at all levels of their organization, and in the first instance to have a clearer picture of the architectures that sustain their dynamics.

In this essay we have shown that the operational functions of basic structures of the IS such as the TLR, the TCR, and the proteasome obey global principles, and are organized according to general architectures and structures that work in a strictly and deeply intertwined manner, such as those of network, degeneracy and bow tie. These are the result of evolutionary processes of optimization between economy of resources and capability of reaction. Indeed, from the viewpoint of ecological immunology, it is assumed that immunological defences must be minimized in terms of cost, i.e. energy expenditure [74,75]. We recently discussed the hypothesis that the bow tie architecture might be suitable for describing the variety of immune-neuroendocrine inputs that continuously target cells and organs while, at the same time, fulfilling the basic requirement of minimizing the cost of immune-neuroendocrine responses [76].

On the other hand, emerging evidence about genetic networks links up wiring patterns of interactions (architecture) with their behaviour in the presence of biological noise, suggesting that noise has a role directly encoded in gene circuit architecture [77].

Recent proposals in the direction of this integrative approach envisage the complex architecture of metabolic pathways as a *network of modular and nested bow ties *[41]. The advantage of this approach is that the elements of the network are no longer considered as simple entities, but rather as functional modular units, interacting on different functional layers and characterized by a sophisticated level of complexity. The drawback of this approach is the intrinsic difficulty of a rigorous (mathematical) tractability, which is a urgent challenge in systems biology [78]. Similarly, a better understanding of proteasome function will be able to overcome the limits of available models [79], which are still unable to account for the full universe of the generated peptides and its dynamics.

In general, we surmise that a systematic and integrative use of concepts such as degeneracy and bow tie architecture, in combination with and within the framework of a network perspective [3], should be very useful not only for elucidating the general rules governing complex biological systems but also for identifying their hidden and specific fragilities and weak points, which represent the start of pathologies, extending previous suggestions from pioneer scientists [5,6,13].

## Competing interests

The authors declare that they have no competing interests.

## Authors' contributions

PT, AG, CF, DR and GCC conceived of the study, AZ and MM participated in its development, PT, AG and CF drafted the manuscript. All authors contributed to write the manuscript, and then read and approved the final manuscript.

## Appendix. History and pervasiveness of degeneracy

### The origin: a physico-mathematical notion

The first use of the term degeneracy in the scientific literature can be traced to the early days of quantum theory when it came to define different stationary states (with different wave-functions) corresponding to the same energy level [80-82]. During the heyday of quantum theory in the 1930s and 1940s, different nascent disciplines began to borrow concepts from physics in an attempt to acquire scientific prestige: Degeneracy was chosen specifically by biology, biochemistry and communication engineering [83,84]. In such new contexts, the notion of degeneracy abandoned its original physico-chemical meaning and came to define any class of objects in which different elements (i.e. inputs) could perform the same function (i.e. output).

### Biological acceptance: the genetic code is degenerate

The first entry of degeneracy into the biological field was due to Crick in 1955 [85]. Inspired by Gamow's reflections on the relationship between DNA and proteins [86], Crick dedicated a paper on genetic code degeneracy, suggesting that its role was essential for explaining how different codons could express one amino acid. Ensuing theoretical analyses stressed the importance of degeneracy by considering Crick's "central dogma" of the unidirectional flow of genetic information (from DNA to RNA to protein [87-89]), "a *purely mathematical property *of the degeneracy of the genetic code" [90].

### Immunological and neural speculations

Although molecular biology favoured the entry of degeneracy into the biological field, immunology has to be recognized as the discipline that gave it a fundamental and wide-ranging explanatory role. In 1959 Talmage opposed the long-lasting "one-antigen, one-antibody" model by introducing the idea that different globulins would cross-react with a single antigen [91], a concept Eisen named degeneracy ten years later [92]. Along these lines of research, Edelman further developed the concept of degeneracy by suggesting two different operative dimensions: (i) at the level of the antibody-gene repertoire, degeneracy was the underlying mechanism used by the IS to achieve both specificity (i.e., self-nonself discrimination, tolerance, booster effect) and universality (i.e., generation of diversity) in antigen recognition; (ii) at the organismal level, and then presuming an analogy between somatic and natural selection mechanisms, degeneracy was also a general evolutionary strategy to produce adaptability to unforeseen environments [93,94].

In his subsequent shift to neurobiology, Edelman explained the synaptic function of the nervous system as similar to the mechanism of cellular differentiation and selection of the IS, a new context in which degeneracy was used to describe two structurally different neural networks equivalent in their abilities to respond to a certain signal [95,96]. The formation of a repertoire of degenerate neuronal groups could then explain the brain as a modular system, affording it an evolutionary robustness to damage via the substitution of the damaged structure by others performing the same function [97,98].

### The pervasive multidisciplinarity of degeneracy

By extending the theory of neuronal group selection to embrace computer modelling, Edelman subsequently used degeneracy to design selective network-based automata to improve their learning and recognition ability [99,100]. In considering degeneracy to be a prominent property of evolution itself - being both a prerequisite for, and an inevitable outcome of, natural selection - Edelman attempted to apply it to a list of 22 phenomena over all levels of biological organization, ranging from the genetic code, through molecular and functional brain architectures, to human communication [4]. To underpin such a large-scale program, he also offered a more coherent formalization and provided a mathematical treatment of degeneracy that turned out to be strictly related to a measure of biological complexity [101,102]. Owing to the inherent stereochemical nature of both antigen-antibody interactions and synaptic networks, Edelman has the theoretical credit of having integrated the classical mathematical framework of degeneracy with a new topobiological interpretation that knits together the structural and functional dimensions of biological organisms.

Recent scientific literature, especially in the immunological and neurological fields, has paid increasing attention to degeneracy as an organizing principle for describing the properties and dynamics of complex biological networks. As for immunology, works worth mentioning analyze degeneracy as: a historical event in the context of antigen-antibody reaction [103]; the "Yin and Yang of the immune system" for its pivotal role in T and B cell functions [104]; an argument in the ongoing debate to discredit self-nonself theory [105]; a tool to design vaccines for the treatment of infectious diseases ([106,107] and cancer [108]; an age-associated parameter of T-cell reactivity [109]; a main property of the IS cognate to, yet different from, that of cross-reactivity [103,110], molecular mimicry [111,112], polyspecificity [38,113], promiscuity [107,110,114], pluripotentiality [25] and specificity [78,115]. Degeneracy is also applied in neuroscience to explain neuroanatomical functional architecture [24-26], in computational biology to improve PCR performance [116,117], in evolutionary biology as a key parameter for evaluating complexity [101,102,118], species evolutionary distance [119] and evolution of morphological novelties [120], as well as in psychology as a mechanism underling language acquisition [121].

We are aware that the historical development of degeneracy dealt with concepts (e.g. modularity and robustness) and biological levels (cellular networks, distributed systems, evolutionary dimension) that are typically involved in bow tie models. Both notions are indeed based on a promising many-to-one structure-function relationship (Figure 2), which seems to be a ubiquitous architectural constraint exploited by evolution to afford efficiency (modular-base structures), robustness (non-catastrophic response to variations) and evolvability in highly complex networks.
